# Axillary Dacron Graft Versus Direct Femoral Arterial Cannulation for Peripheral VA-ECMO After Heart Transplantation: A Single-Center Cohort Study

**DOI:** 10.3390/biomedicines14102408

**Published:** 2026-10-21

**Authors:** Lorenzo Giovannico, Giuseppe Fischetti, Domenico Parigino, Luca Savino, Claudia Leo, Giuseppe Cristiano, Ilaria Paradiso, Federica Ferrara, Francesca Broggini, Carlo Mancini, Massimiliano Carrozzini, Massimo Padalino, Tomaso Bottio

**Affiliations:** Cardiac Surgery Unit, Department of Precision and Regenerative Medicine and Ionian Area (DiMePRe-J), University of Bari Aldo Moro, Piazza Giulio Cesare 11, 70124 Bari, Italy

**Keywords:** venoarterial extracorporeal membrane oxygenation (VA-ECMO), primary graft dysfunction, heart transplantation, axillary artery cannulation, femoral artery cannulation

## Abstract

**Background/Objectives:** Primary graft dysfunction (PGD) remains a major cause of early morbidity and mortality after heart transplantation, and peripheral veno-arterial extracorporeal membrane oxygenation (VA-ECMO) represents an established rescue therapy for severe cases. Although direct femoral arterial cannulation is commonly used because of its rapid deployment, axillary artery cannulation through a Dacron graft provides antegrade arterial perfusion and may represent an alternative approach. This study compared early clinical outcomes and cannulation-related complications between these two arterial cannulation strategies. **Methods:** In this retrospective single-center observational study, 42 consecutive adult heart transplant recipients requiring peripheral VA-ECMO for PGD were included. Patients were divided according to arterial cannulation strategy into direct femoral cannulation (DFC, *n* = 25) and axillary Dacron graft cannulation (ADG, *n* = 17). Baseline recipient and donor characteristics, perioperative variables, postoperative outcomes, vascular complications, successful VA-ECMO weaning, and survival to hospital discharge were compared. Odds ratios (ORs) with 95% confidence intervals (CIs) were calculated for major clinical outcomes and cannulation-related complications. **Results:** The primary composite endpoint of cannulation-related complications occurred in 0/17 patients (0%) in the ADG group and 3/25 patients (12.0%) in the DFC group (OR 0.18, 95% CI 0.01–3.80; *p* = 0.260). Successful VA-ECMO weaning was achieved in 94.1% versus 92.0% (*p* = 1.000), and survival to hospital discharge was 76.5% versus 64.0% (OR 1.83, 95% CI 0.46–7.32; *p* = 0.505). Stroke occurred in 5.9% versus 16.0% (OR 0.33, 95% CI 0.03–3.23; *p* = 0.632), while renal replacement therapy was required in 35.3% versus 52.0% (OR 0.50, 95% CI 0.14–1.79; *p* = 0.353). No statistically significant differences were detected between groups. **Conclusions:** No statistically significant differences in successful VA-ECMO weaning or survival to hospital discharge were observed between axillary Dacron graft and direct femoral arterial cannulation. No statistically significant differences in neurological, vascular, or other major postoperative outcomes were detected. Although several complications occurred numerically less frequently with axillary cannulation, the limited sample size and wide confidence intervals preclude conclusions regarding comparative superiority. Axillary Dacron graft cannulation may therefore represent a feasible alternative in selected heart transplant recipients requiring peripheral VA-ECMO for PGD. Larger prospective multicenter studies are warranted.

## 1. Introduction

Primary graft dysfunction (PGD) remains the leading cause of early morbidity and mortality after heart transplantation despite continuous advances in donor selection, organ preservation, and perioperative management [1,2]. Moreover, the increasing use of extended-criteria donors to expand the available donor pool may expose recipients to a higher risk of primary graft dysfunction (PGD), particularly when multiple donor-related risk factors coexist [3,4,5,6].

Severe PGD is characterized by profound ventricular dysfunction occurring within the first hours after transplantation and frequently requires temporary mechanical circulatory support to maintain adequate systemic perfusion while allowing myocardial recovery.

Peripheral veno-arterial extracorporeal membrane oxygenation (VA-ECMO) has become the preferred rescue strategy for severe PGD, providing immediate cardiopulmonary support, maintaining systemic perfusion, and allowing time for myocardial recovery [2]. Early initiation has been associated with improved myocardial recovery and survival compared with delayed implantation. While severe PGD remains a major indication for VA-ECMO in the heart transplant setting, its clinical application extends to a broad spectrum of acute cardiovascular conditions, including refractory cardiogenic shock, acute myocardial infarction, fulminant myocarditis, refractory ventricular arrhythmias, massive pulmonary embolism, and postcardiotomy shock. Moreover, VA-ECMO may serve as a bridge to myocardial recovery, durable mechanical circulatory support, or heart transplantation [2,7,8,9,10].

Although direct femoral arterial cannulation remains the most widely adopted approach because of its rapid accessibility and technical simplicity, it may be associated with vascular complications including lower-limb ischemia, arterial injury, wound infection, bleeding, and retrograde aortic perfusion [11,12,13,14]. To overcome these limitations, axillary artery cannulation through a side Dacron graft has emerged as an attractive alternative, providing antegrade systemic perfusion while potentially reducing access-related complications and facilitating prolonged extracorporeal support.

Previous studies comparing axillary and femoral arterial cannulation have mainly included heterogeneous populations with cardiogenic shock from different etiologies, whereas evidence focusing specifically on heart transplant recipients with PGD remains limited [11,12,13,14]. Whether graft-mediated axillary cannulation provides clinically relevant advantages over direct femoral cannulation in this highly selected population therefore remains uncertain.

The aim of this study was to compare early clinical outcomes, successful ECMO weaning, hospital survival, and cannulation-related complications between axillary Dacron graft cannulation and direct femoral arterial cannulation in adult heart transplant recipients requiring peripheral VA-ECMO for severe PGD.

## 2. Materials and Methods

### 2.1. Study Design and Patient Population

**Figure 1:**
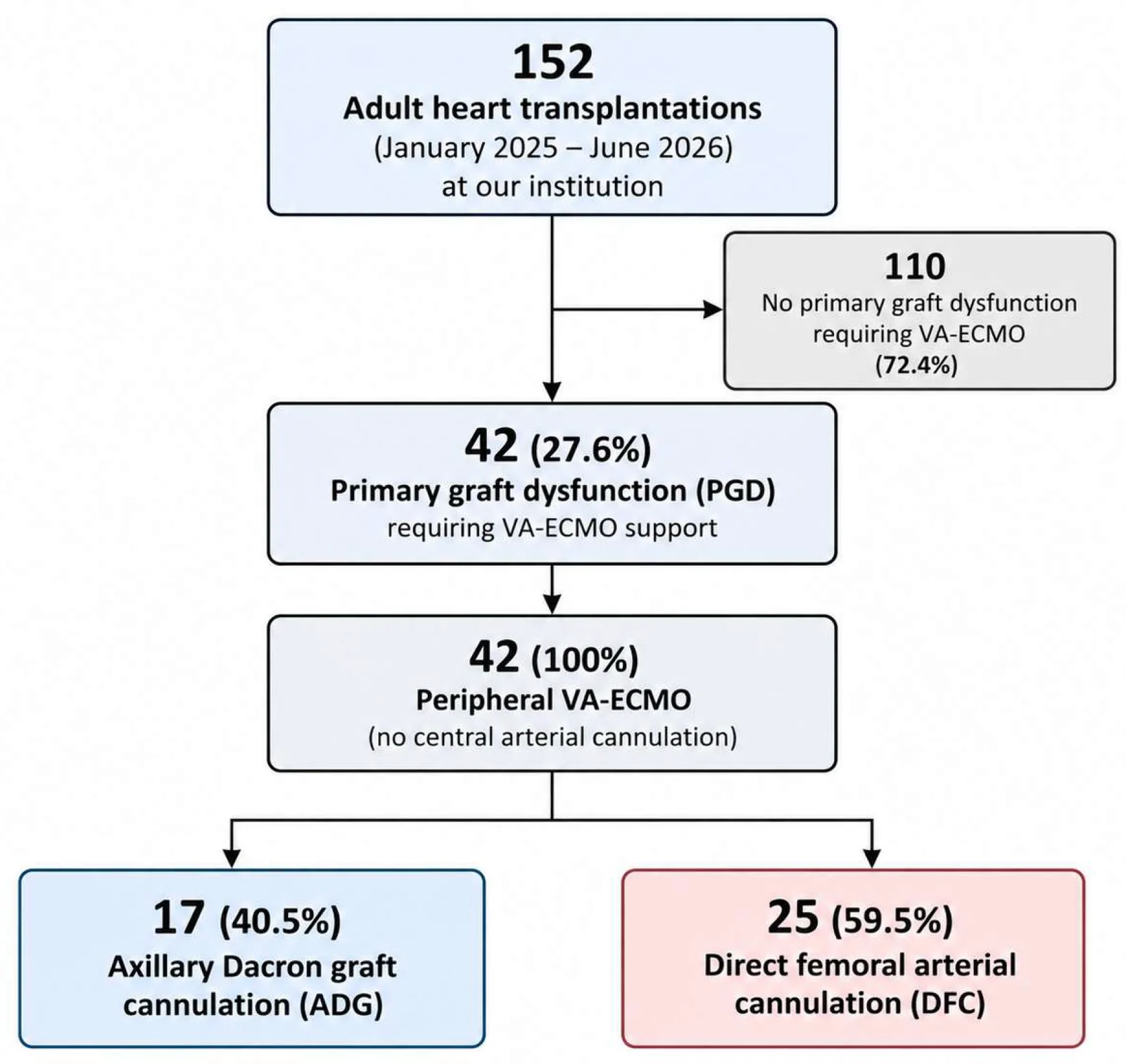
Patient flow diagram. Flow of patients through the study, from total heart transplantations to the final study groups. PGD, primary graft dysfunction; VA-ECMO, veno-arterial extracorporeal membrane oxygenation; ADG, axillary Dacron graft; DFC, direct femoral arterial cannulation.

This retrospective, single-center observational study included all consecutive adult patients who underwent peripheral veno-arterial extracorporeal membrane oxygenation (VA-ECMO) for primary graft dysfunction (PGD) following orthotopic heart transplantation at the Cardiac Surgery Unit, University of Bari Aldo Moro, between January 2025 and June 2026. During the study period, 152 adult patients underwent orthotopic heart transplantation at our institution. Among these, 42 patients (27.6%) developed PGD requiring VA-ECMO support. All 42 patients were managed with peripheral VA-ECMO; no patient requiring VA-ECMO for PGD underwent central arterial cannulation. Therefore, all 42 consecutive patients were included in the present analysis, with 17 undergoing axillary Dacron graft cannulation and 25 undergoing direct femoral arterial cannulation (Figure 1).

Primary graft dysfunction was diagnosed according to the International Society for Heart and Lung Transplantation (ISHLT) consensus definition. Twenty-four of the 42 patients (57.1%) had been supported with VA-ECMO before transplantation as a bridge to transplantation (9/17 in the ADG group and 15/25 in the DFC group). During heart transplantation, all patients were managed with conventional cardiopulmonary bypass, and separation from CPB was attempted after graft implantation and reperfusion. In all patients included in the present cohort, adequate graft function and hemodynamic stability could not be achieved despite optimization of medical therapy, requiring transition from CPB to peripheral VA-ECMO. Thus, in patients receiving pre-transplant VA-ECMO, postoperative ECMO support was not considered merely a continuation of pre-transplant support but was instituted after unsuccessful separation from CPB because of inadequate early allograft function. No patient underwent de novo VA-ECMO implantation later during the postoperative course.

Patients were divided into two groups according to the arterial cannulation strategy used for VA-ECMO implantation:

- Direct Femoral Cannulation (DFC), in which the arterial cannula was inserted directly into the common femoral artery.
- Axillary Dacron Graft Cannulation (ADG), in which arterial inflow was established through an end-to-side Dacron graft anastomosed to the right axillary artery.

The study was conducted in accordance with the Declaration of Helsinki and approved by the Institutional Ethics Committee. Written informed consent was obtained from all patients prior to inclusion in the study.

### 2.2. Surgical Technique

Peripheral VA-ECMO was implanted by experienced cardiac surgeons according to institutional protocols. In the direct femoral cannulation group, the common femoral artery was surgically exposed, and a 16- or 17-Fr arterial ECMO cannula was inserted directly into the vessel. Distal limb perfusion was systematically established in all 25 patients (100%) using a 7-Fr reperfusion cannula inserted into the superficial femoral artery using the Seldinger technique. Lower-limb perfusion was continuously monitored bilaterally using near-infrared spectroscopy (NIRS; INVOS™), allowing comparison between the cannulated and contralateral limbs throughout VA-ECMO support. In the axillary group, the right axillary artery was exposed through an infraclavicular incision. After systemic heparinization, an 8 mm Dacron vascular graft, approximately 4–6 cm in length, was anastomosed end-to-side to the artery using continuous 5-0 polypropylene sutures. A 16- or 17-Fr arterial ECMO cannula was then inserted into the graft rather than directly into the native vessel, allowing antegrade systemic perfusion while preserving distal upper-limb blood flow. In both groups, venous drainage was obtained through the common femoral vein using a 21-Fr multistage venous cannula positioned under transesophageal echocardiographic guidance.

At VA-ECMO decannulation, the arterial cannula was surgically removed, and the native artery was explored using a Fogarty catheter to remove any residual thrombotic material or intraluminal clot, when present. Surgical arteriorrhaphy was subsequently performed to restore arterial integrity. This approach was applied to both the femoral artery in the DFC group and the axillary artery in the ADG group.

The choice of arterial cannulation strategy depended on surgeon preference, patient anatomy, anticipated duration of support, and the presence of peripheral vascular disease or contraindications to femoral access.

VA-ECMO management followed our previously described institutional protocol [10]. Partial extracorporeal support was generally maintained at a target flow of 2.2–3.0 L/min/m^2^ and adjusted according to systemic perfusion, native cardiac function, and hemodynamic requirements. Unfractionated heparin was initiated 24 h after sternal closure, once ongoing surgical bleeding had been excluded, at an initial infusion rate of 400 U/h. Anticoagulation was monitored using activated clotting time (ACT) every 4 h, with a target of 180–200 s, and activated partial thromboplastin time (aPTT) every 8 h, with a target of 45–60 s. Left ventricular unloading was considered in the presence of elevated pulmonary capillary wedge pressure, left ventricular distension with impaired contractility and blood stasis, persistent aortic valve closure, pulmonary congestion, or ventricular arrhythmias. Unloading strategies included optimization of inotropic therapy and, when clinically indicated, IABP, Impella, or surgical ventricular venting.

Assessment for VA-ECMO weaning was generally initiated after 48–72 h of support. Weaning was considered in hemodynamically stable patients with MAP > 60 mmHg, PCWP < 18 mmHg, cardiac index > 2.4 L/min/m^2^, SvO_2_ ≥ 70%, LVEF ≥ 25%, preserved right ventricular function, lactate < 2 mmol/L, and adequate urine output or renal replacement therapy. ECMO flow was progressively reduced under echocardiographic and hemodynamic monitoring, followed by a low-flow trial before decannulation.

### 2.3. Data Collection

Clinical data were retrospectively extracted from the institutional electronic medical records.

Baseline recipient variables included the following:

- Age;
- Body surface area;
- Ischemic versus non-ischemic cardiomyopathy;
- Previous cardiac surgery;
- LVAD support;
- Implantable cardioverter-defibrillator;
- Diabetes mellitus;
- Hypertension;
- Peripheral vascular disease;
- Cerebrovascular disease;
- Smoking history;
- Pre-transplant mechanical circulatory support (IABP, Impella, ECMO);
- Preoperative renal replacement therapy.

Donor characteristics included the following:

- Donor age;
- Donor BSA;
- Donor LVEF;
- Ischemic time;
- Cardiopulmonary bypass time.

Postoperative variables included ECMO duration, ICU stay, hospital stay, successful ECMO weaning, renal replacement therapy, mechanical ventilation duration, tracheostomy, neurological events, bleeding requiring re-exploration, vascular complications, limb ischemia, upper extremity ischemia, wound complications, and in-hospital mortality. No missing data were present for the variables included in the comparative analyses.

### 2.4. Study Endpoints

The primary endpoint was the occurrence of major cannulation-related vascular complications, defined as follows:

- Limb ischemia requiring intervention;
- Upper extremity ischemia;
- Surgical site bleeding requiring re-exploration;
- Vascular injury;
- Wound infection;
- Pseudoaneurysm;
- Need for surgical revision of the cannulation site.

Secondary endpoints included the following:

- Successful ECMO weaning;
- Stroke;
- Tracheostomy;
- Renal replacement therapy;
- In-hospital mortality.

Additional exploratory outcomes included prolonged mechanical ventilation, defined as invasive mechanical ventilation for ≥7 days, bleeding requiring surgical re-exploration, and gastrointestinal bleeding. De novo postoperative RRT was evaluated as a sensitivity analysis after excluding patients receiving RRT before transplantation.

### 2.5. Definitions

PGD severity was adjudicated according to the ISHLT consensus criteria. Because all patients included in the present cohort required VA-ECMO for PGD, all fulfilled the criteria for severe PGD according to the ISHLT classification.

Successful ECMO weaning was defined as survival for more than 24 h after decannulation without reinstitution of mechanical circulatory support.

Acute kidney injury requiring continuous renal replacement therapy was defined according to KDIGO criteria. The overall postoperative RRT endpoint included patients already receiving continuous renal replacement therapy before transplantation. A sensitivity analysis was therefore performed after excluding patients receiving preoperative RRT to assess de novo postoperative RRT.

Stroke was defined as any new focal neurological deficit confirmed by computed tomography.

Major bleeding was defined as bleeding requiring surgical re-exploration.

Vascular complications comprised limb ischemia, upper-limb ischemia, arterial thrombosis, pseudoaneurysm, wound infection, or bleeding requiring operative management. Postoperative and cannulation-related complications were assessed throughout the entire postoperative hospitalization, from VA-ECMO initiation until the first hospital discharge following heart transplantation. Accordingly, access-related complications occurring after VA-ECMO decannulation but before the first post-transplant hospital discharge were included in the analysis.

### 2.6. Statistical Analysis

Continuous variables were assessed for normality using the Shapiro–Wilk test and are reported as mean ± standard deviation (SD) or median with interquartile range (IQR), as appropriate.

Categorical variables are expressed as frequencies and percentages.

Baseline characteristics and postoperative outcomes were compared between the two groups using Student’s *t* test or Mann–Whitney *U* test for continuous variables and the χ^2^ test or Fisher’s exact test for categorical variables.

Odds ratios (ORs) with 95% confidence intervals (CIs) were calculated for major postoperative complications. All odds ratios were calculated using the ADG group as the exposed group and the DFC group as the reference group (OR > 1 indicating higher odds of the outcome in the ADG group). Absolute risk differences (RDs) with 95% confidence intervals were also calculated for clinical outcomes. Risk differences were defined as the event rate in the axillary group minus that in the femoral group, with 95% confidence intervals estimated using the Newcombe–Wilson method. For 2 × 2 tables containing a zero cell, ORs and corresponding 95% CIs were estimated using a 0.5 continuity correction; ORs were not estimated when no events occurred in either group.

A two-sided *p*-value < 0.05 was considered statistically significant.

Statistical analyses were performed using IBM SPSS Statistics (IBM SPSS Statistics 32.0, IBM Corp., Armonk, NY, USA). No formal sample-size calculation was performed because the study included the entire consecutive cohort of eligible patients during the predefined study period. Given the limited sample size and number of events, all between-group comparisons were considered exploratory. No adjustment for multiple comparisons was performed, and effect estimates with 95% confidence intervals were emphasized rather than post hoc power calculations.

## 3. Results

### 3.1. Patient Characteristics

During the study period, 152 adult heart transplantations were performed. PGD requiring VA-ECMO occurred in 42 recipients (27.6%). All 42 patients received peripheral VA-ECMO and constituted the final study cohort. Of these, 17 (40.5%) underwent arterial cannulation through a right axillary Dacron graft and 25 (59.5%) underwent direct femoral arterial cannulation. Twenty-four patients (57.1%) were already supported with VA-ECMO before transplantation, whereas the remaining 18 (42.9%) were transitioned directly from cardiopulmonary bypass to VA-ECMO at the end of transplantation. No patient required de novo VA-ECMO initiation later in the postoperative course. Baseline recipient and donor characteristics are summarized in Table 1. No statistically significant between-group differences were detected for the evaluated baseline variables. However, numerical differences were observed for some characteristics, including recipient age, previous cardiac surgery, peripheral vascular disease, and preoperative ECMO support. Given the limited sample size and the non-randomized selection of cannulation strategy, the absence of statistically significant differences should not be interpreted as evidence of baseline equivalence.

### 3.2. Operative Characteristics

Operative and donor-related variables are presented in Table 2. Median donor heart ischemic time was 220.0 (198.0–245.0) min in the axillary group and 240.0 (223.0–250.0) min in the femoral group (*p* = 0.327), while median cardiopulmonary bypass duration was 276.0 (223.0–290.0) and 223.0 (204.0–249.0) min, respectively (*p* = 0.115). Median VA-ECMO support duration was 5.0 (4.0–5.0) days in both groups (*p* = 0.641). No statistically significant between-group differences were detected for pre-transplant cardiogenic shock or donor infection.

### 3.3. Early Clinical Outcomes

Clinical outcomes are summarized in Table 3.

Successful VA-ECMO weaning was achieved in 16/17 patients (94.1%) in the axillary group and 23/25 (92.0%) in the femoral group (*p* = 1.000). Of the 42 patients included in the study, 39 (92.9%) were successfully weaned from VA-ECMO, whereas three patients died without successful ECMO weaning. Among the 39 successfully decannulated patients, 29 survived to hospital discharge, while 10 died later during the postoperative course. The median interval from VA-ECMO decannulation to death among these patients was 29 days (IQR 21–36; range 9–60 days). All post-decannulation deaths were attributable to multiorgan failure rather than recurrent cardiac or graft failure. Importantly, none of the patients successfully weaned from VA-ECMO required reinstitution of VA-ECMO or any other form of temporary mechanical circulatory support before hospital discharge. Survival to hospital discharge was 13/17 (76.5%) and 16/25 (64.0%), respectively (OR 1.83, 95% CI 0.46–7.32; *p* = 0.505). Stroke occurred in 1/17 patients (5.9%) in the axillary group and 4/25 (16.0%) in the femoral group (OR 0.33, 95% CI 0.03–3.23; *p* = 0.632). Tracheostomy was required in 0/17 and 4/25 patients (16.0%), respectively (*p* = 0.134). Renal replacement therapy was required in 6/17 patients (35.3%) in the axillary group and 13/25 (52.0%) in the femoral group (OR 0.50, 95% CI 0.14–1.79; *p* = 0.353). Of these patients, one in the ADG group and three in the DFC group were already receiving continuous renal replacement therapy before transplantation and were therefore included in the overall RRT endpoint. In a sensitivity analysis restricted to patients not receiving preoperative RRT, de novo postoperative RRT was required in 5/16 patients (31.3%) in the ADG group and 10/22 (45.5%) in the DFC group (*p* = 0.506). Prolonged mechanical ventilation ≥ 7 days occurred in 1/17 (5.9%) and 2/25 (8.0%) patients, respectively (*p* = 1.000). None of these between-group differences reached statistical significance.

### 3.4. Cannulation-Related Complications

Cannulation-related complications are reported in Table 3. No arterial cannulation-site bleeding occurred in the axillary group, whereas this event was recorded in 3/25 patients (12.0%) in the femoral group. Wound infection occurred in 0/17 and 3/25 patients (12.0%), respectively. The three patients in the femoral group who experienced cannulation-site bleeding were the same patients who subsequently developed wound infection. Accordingly, each patient was counted only once in the composite primary endpoint, resulting in an overall incidence of cannulation-related complications of 3/25 (12.0%) in the DFC group and 0/17 (0%) in the ADG group. Importantly, none of the surgical re-explorations for bleeding in the ADG group originated from the axillary cannulation site or Dacron graft anastomosis. These events were therefore classified as postoperative bleeding complications but were not included in the cannulation-related primary composite endpoint. No upper- or lower-limb ischemia or limb hyperperfusion was observed in either group.

Bleeding requiring surgical intervention occurred in 4/17 patients (23.5%) in the axillary group and 1/25 (4.0%) in the femoral group (OR 7.38, 95% CI 0.75–73.14; *p* = 0.140). None of the observed between-group differences in individual vascular or bleeding outcomes reached statistical significance.

### 3.5. Exploratory Analysis

Forest plot analysis demonstrated wide 95% confidence intervals for most effect estimates, with the intervals crossing unity for all evaluated outcomes. Numerical differences between groups were observed for several endpoints, including stroke, tracheostomy, renal replacement therapy, arterial cannulation-site bleeding, wound infection, and bleeding requiring surgical intervention. However, none of these differences reached statistical significance. Given the small sample size and low number of events, these estimates should be considered exploratory and interpreted with caution.

## 4. Discussion

The optimal arterial cannulation strategy for peripheral VA-ECMO remains a matter of ongoing debate, particularly in heart transplant recipients who develop primary graft dysfunction (PGD). Although femoral cannulation is widely adopted because of its rapid accessibility and technical simplicity, axillary cannulation through a prosthetic Dacron graft represents an alternative approach that provides antegrade systemic perfusion and avoids direct femoral arterial cannulation. However, comparative evidence in the specific setting of heart transplantation remains limited.

Beyond the specific setting of post-transplant PGD, VA-ECMO represents an established form of temporary mechanical circulatory support across a broad spectrum of life-threatening cardiovascular conditions. Its indications include refractory cardiogenic shock secondary to acute myocardial infarction, fulminant myocarditis, and acute decompensation of end-stage heart failure, as well as selected cases of refractory ventricular arrhythmias in which extracorporeal support may provide hemodynamic stability during complex catheter ablation. VA-ECMO may also be considered in massive pulmonary embolism with circulatory collapse and, in carefully selected patients, in severe sepsis-associated cardiovascular failure. Importantly, VA-ECMO may serve not only as a bridge to myocardial recovery or clinical decision-making but also as a bridge to durable ventricular assist device implantation, total artificial heart, or heart transplantation [7,8,9,10]. In this broader clinical context, arterial cannulation strategy should be individualized according to the urgency of support, expected ECMO duration, vascular anatomy, and anticipated subsequent therapeutic pathway.

In the present cohort, no statistically significant differences were detected between axillary Dacron graft and direct femoral cannulation in successful ECMO weaning, survival to hospital discharge, neurological complications, renal replacement therapy, or the other evaluated early clinical outcomes. Successful ECMO weaning occurred in 94.1% and 92.0% of patients, respectively, while survival to hospital discharge was 76.5% and 64.0%. Although numerical differences were observed for several secondary outcomes, including stroke, tracheostomy, renal replacement therapy, arterial cannulation-site bleeding, and wound infection, all corresponding comparisons were non-significant, and the confidence intervals around the effect estimates were wide. These findings should therefore be interpreted as descriptive and exploratory rather than as evidence of a comparative benefit of either cannulation strategy.

With regard to local access-related events, no arterial cannulation-site bleeding or wound infection was observed in the axillary group, whereas each occurred in three patients (12.0%) undergoing direct femoral cannulation. However, these differences did not reach statistical significance. The anatomical and technical characteristics of the two approaches may nevertheless be relevant when selecting arterial access. Direct femoral cannulation involves instrumentation of the groin and retrograde arterial perfusion, whereas an end-to-side axillary Dacron graft provides antegrade arterial inflow without direct insertion of a large-bore cannula into the native axillary artery. The present data are insufficient to determine whether these technical differences translate into a lower incidence of access-related complications.

Our observations can be considered in the context of previous studies comparing axillary and femoral VA-ECMO access. Ohira et al. reported comparable survival between axillary and femoral VA-ECMO and differences in wound-related and limb complications between approaches [14]. More importantly, in a subsequent study specifically evaluating heart transplant recipients requiring VA-ECMO for PGD, the same group reported differences in cannulation-site complications according to arterial access without compromising successful ECMO weaning or survival. Given the limited comparative evidence specifically involving heart transplant recipients, our study provides additional single-center data in this selected population.

Similarly, Pisani et al. reported their experience with right axillary artery cannulation through a Dacron side graft, including low incidences of local bleeding, upper-limb ischemia, wound infection, and brachial plexus injury [15]. Chamogeorgakis et al. also described axillary side-graft cannulation as a feasible approach for peripheral ECMO support [16]. Nevertheless, differences in patient selection, indications for VA-ECMO, cannulation techniques, and perioperative management limit direct comparison across studies, and the available evidence does not establish the superiority of one arterial access strategy.

Neurological injury remains one of the most important complications of VA-ECMO. In our series, stroke occurred in 5.9% of patients undergoing axillary cannulation and 16.0% of those undergoing femoral cannulation; however, this difference was not statistically significant (OR 0.33, 95% CI 0.03–3.23; *p* = 0.632). Vale et al. reported a different distribution of neurological events according to ECMO configuration [17], whereas Nishikawa et al. found no association between arterial cannulation site and stroke incidence [18]. Neurological complications during VA-ECMO are multifactorial and may be influenced by patient characteristics, embolic burden, anticoagulation management, underlying cardiac pathology, and duration of extracorporeal support. Accordingly, the numerical difference observed in our cohort cannot be attributed to the cannulation strategy.

No upper- or lower-limb ischemic events were observed in either group. Femoral arterial cannulation has historically been associated with lower-limb ischemia, particularly in the absence of distal limb perfusion. Importantly, distal limb perfusion was systematically established in all patients undergoing direct femoral cannulation using a 7-Fr reperfusion cannula placed in the superficial femoral artery. This standardized approach may have contributed to the absence of clinically detected lower-limb ischemic complications, although the small sample size precludes definitive conclusions regarding ischemic risk. Tellioglu et al. similarly reported no significant difference in vascular complications between femoral and axillary cannulation in a contemporary VA-ECMO cohort [19]. These findings highlight the importance of standardized distal perfusion strategies when femoral arterial cannulation is used.

Regardless of arterial cannulation strategy, comprehensive cardiovascular monitoring remains essential during VA-ECMO support. The interaction between native cardiac output and extracorporeal blood flow generates a dynamic hemodynamic condition in which arterial pressure alone may provide an incomplete assessment of cardiovascular performance. Clinical examination, invasive arterial pressure monitoring, central venous pressure, serial echocardiography, blood-gas analysis, lactate kinetics, and, when appropriate, advanced hemodynamic monitoring should therefore be integrated to assess systemic perfusion, ventricular loading conditions, and myocardial recovery. Particular attention should be paid to left ventricular distension, aortic valve opening, pulmonary congestion, and the balance between native and extracorporeal flow. Moreover, peripheral VA-ECMO may generate differential oxygenation according to the relative contribution of native cardiac output and extracorporeal flow. A thorough understanding of these physiological interactions is essential for the correct interpretation of hemodynamic measurements and for tailoring ECMO flow, ventricular unloading, vasoactive therapy, and ultimately the timing of weaning [20].

Pharmacological management represents another important component of VA-ECMO care and should be considered alongside the technical aspects of extracorporeal support. Vasoactive and inotropic agents are frequently required to maintain adequate perfusion pressure and support residual myocardial function; however, excessive catecholamine exposure may increase myocardial oxygen consumption, arrhythmic burden, and peripheral vasoconstriction. Pharmacological therapy should therefore be individualized according to the underlying shock phenotype, native ventricular function, ECMO flow, and end-organ perfusion. In addition, VA-ECMO may alter drug pharmacokinetics through changes in volume of distribution, organ perfusion, and sequestration within the extracorporeal circuit. These considerations are particularly relevant in heart transplant recipients with severe PGD, in whom vasoactive therapy, immunosuppression, antimicrobial treatment, anticoagulation, and extracorporeal support frequently coexist. Accordingly, cannulation strategy represents only one component of a broader multimodal management approach [21].

An additional observation was the higher numerical incidence of bleeding requiring surgical intervention in the axillary group (23.5% vs. 4.0%; OR 7.38, 95% CI 0.75–73.14; *p* = 0.140). This difference was not statistically significant and, given the small number of events, no causal relationship with the cannulation technique can be established. Axillary graft implantation requires surgical exposure, arterial dissection, and creation of a prosthetic anastomosis, which represent additional potential bleeding sites. However, detailed attribution of bleeding events is necessary before considering these events directly related to arterial access. This observation further emphasizes that the potential advantages and disadvantages of each technique should be considered individually.

An important consideration when interpreting these findings is the potential for confounding by indication. Cannulation strategy was not randomly assigned but reflected surgeon preference, anatomical suitability, clinical urgency, anticipated duration of extracorporeal support, and contraindications to femoral access. Consequently, patients selected for axillary cannulation may have differed in clinically relevant measured or unmeasured characteristics from those undergoing direct femoral cannulation. Furthermore, the absence of statistically significant differences in baseline characteristics should not be interpreted as evidence of equivalence between groups, particularly given the limited sample size.

Taken together, our findings do not demonstrate superiority or equivalence of either cannulation strategy. Rather, they show that axillary Dacron graft cannulation was feasible in this selected cohort and was associated with observed rates of ECMO weaning and survival within the range observed with direct femoral cannulation. The choice of arterial access should therefore remain individualized according to clinical urgency, vascular anatomy, anticipated duration of support, and institutional expertise. Larger prospective multicenter studies with adequate statistical power and standardized definitions of cannulation-related complications are required to determine whether clinically meaningful differences exist between these strategies.

## 5. Conclusions

No statistically significant differences in successful VA-ECMO weaning or survival to hospital discharge were observed between axillary Dacron graft cannulation and direct femoral arterial cannulation in this single-center cohort of heart transplant recipients with PGD. No statistically significant differences in neurological, vascular, or other major postoperative complications were detected between strategies. Although some complications occurred numerically less frequently with axillary cannulation, the small sample size and wide confidence intervals preclude conclusions regarding comparative superiority. Axillary graft cannulation may therefore represent a feasible alternative in selected patients, while larger prospective multicenter studies are required to determine whether either strategy confers clinically meaningful advantages.

## 6. Limitations

This study has several limitations. First, its retrospective single-center design limits the generalizability of the findings and introduces the possibility of selection bias. Second, the relatively small sample size and low number of outcome events reduced statistical power, resulting in wide confidence intervals and limiting the ability to detect statistically significant differences between groups. Third, the choice of arterial cannulation strategy was left to surgeon preference rather than random allocation; therefore, residual confounding by indication cannot be excluded despite the absence of significant baseline differences. Finally, only early postoperative outcomes were evaluated, whereas long-term vascular, neurological, graft, and survival outcomes were not available. Consequently, the present findings should be considered exploratory and hypothesis-generating. Larger prospective multicenter studies are required to determine whether axillary Dacron graft cannulation provides clinically meaningful advantages over direct femoral arterial cannulation in heart transplant recipients requiring VA-ECMO for severe primary graft dysfunction.

## Figures and Tables

**Table 1:** Baseline characteristics by cannulation site.

Variable	Axillary (*n* = 17)	Femoral (*n* = 25)	*p* Value	Test
Continuous variables, median (IQR)				Mann–Whitney *U*
Recipient age, years	65.0 (58.0–70.0)	58.0 (51.0–66.0)	0.111	Mann–Whitney *U*
Recipient BSA, m^2^	1.8 (1.7–2.0)	1.8 (1.7–2.0)	0.928	Mann–Whitney *U*
Donor age, years	58.0 (49.0–69.0)	61.0 (52.0–67.0)	0.847	Mann–Whitney *U*
Donor BSA, m^2^	1.8 (1.6–2.1)	1.7 (1.6–2.0)	0.904	Mann–Whitney *U*
Donor LVEF, %	58.5 (50.0–60.0)	60.0 (55.0–65.0)	0.189	Mann–Whitney *U*
Categorical variables, *n*/*N* (%)				Fisher’s exact
Male sex	14/17 (82.4)	17/25 (68.0)	0.477	Fisher’s exact
Non-ischemic cardiomyopathy	11/17 (64.7)	18/25 (72.0)	0.738	Fisher’s exact
Prior cardiac surgery	7/17 (41.2)	6/25 (24.0)	0.314	Fisher’s exact
LVAD	1/17 (5.9)	0/25 (0.0)	0.405	Fisher’s exact
Implantable cardioverter-defibrillator	10/17 (58.8)	18/25 (72.0)	0.508	Fisher’s exact
Diabetes mellitus	5/17 (29.4)	8/25 (32.0)	1.000	Fisher’s exact
Hypertension	12/17 (70.6)	21/25 (84.0)	0.446	Fisher’s exact
Cerebrovascular disease	0/17 (0.0)	0/25 (0.0)	1.000	Fisher’s exact
CVVH pre-ECMO	1/17 (5.9)	3/25 (12.0)	0.635	Fisher’s exact
Peripheral vascular disease	7/17 (41.2)	6/25 (24.0)	0.314	Fisher’s exact
Smoking history	4/17 (23.5)	4/25 (16.0)	0.694	Fisher’s exact
IABP	1/17 (5.9)	1/25 (4.0)	1.000	Fisher’s exact
Impella	1/17 (5.9)	1/25 (4.0)	1.000	Fisher’s exact
ECMO pre-op	9/17 (52.9)	15/25 (60.0)	0.755	Fisher’s exact

**Table 2:** Operative and donor characteristics.

Variable	Axillary (*n* = 17)	Femoral (*n* = 25)	*p* Value	Test
Continuous variables, median (IQR)				Mann–Whitney *U*
Donor heart ischemic time, min	220.0 (198.0–245.0)	240.0 (223.0–250.0)	0.327	Mann–Whitney *U*
Cardiopulmonary bypass time, min	276.0 (223.0–290.0)	223.0 (204.0–249.0)	0.115	Mann–Whitney *U*
Length of ECMO support, days	5.0 (4.0–5.0)	5.0 (4.0–5.0)	0.641	Mann–Whitney *U*
Categorical variables, *n*/*N* (%)				Fisher’s exact
Pre-transplant cardiogenic shock	2/17 (11.8)	8/25 (32.0)	0.162	Fisher’s exact
Donor infection	5/17 (29.4)	3/25 (12.0)	0.235	Fisher’s exact

**Table 3:** Postoperative Outcomes and Cannulation-Related Complications According to VA-ECMO Cannulation Strategy.

Outcome	ADG	DFC	OR (95% CI), ADG vs. DFC	Absolute RD, % (95% CI)	*p* Value
ECMO and survival outcomes					
Successful VA-ECMO weaning	16/17 (94.1%)	23/25 (92.0%)	1.39 (0.12–16.68)	+2.1 (−19.8 to +19.8)	1.000
Survival to hospital discharge	13/17 (76.5%)	16/25 (64.0%)	1.83 (0.46–7.32)	+12.5 (−16.0 to +36.4)	0.505
Major postoperative outcomes					
Stroke	1/17 (5.9%)	4/25 (16.0%)	0.33 (0.03–3.23)	−10.1 (−29.4 to +13.1)	0.632
Prolonged mechanical ventilation ≥ 7 days	1/17 (5.9%)	2/25 (8.0%)	0.72 (0.06–8.62)	−2.1 (−19.8 to +19.8)	1.000
Tracheostomy	0/17 (0%)	4/25 (16.0%)	0.14 (0.01–2.71)	−16.0 (−34.7 to +4.8)	0.134
Postoperative RRT	6/17 (35.3%)	13/25 (52.0%)	0.50 (0.14–1.79)	−16.7 (−42.1 to +13.1)	0.353
De novo postoperative RRT	5/16 (31.3%)	10/22 (45.5%)	0.55 (0.14–2.10)	−14.2 (−40.4 to +16.4)	0.506
Bleeding outcomes					
Any bleeding requiring surgical re-exploration	4/17 (23.5%)	1/25 (4.0%)	7.38 (0.75–73.13)	+19.5 (−1.4 to +43.5)	0.140
Gastrointestinal bleeding	0/17 (0%)	0/25 (0%)	NE	0.0 (−13.3 to +18.4)	1.000
Cannulation-related outcomes					
Cannulation-related complications ‡	0/17 (0%)	3/25 (12.0%)	0.18 (0.01–3.80)	−12.0 (−30.0 to +8.0)	0.260
Arterial cannulation-site bleeding	0/17 (0%)	3/25 (12.0%)	0.18 (0.01–3.80)	−12.0 (−30.0 to +8.0)	0.260
Wound infection	0/17 (0%)	3/25 (12.0%)	0.19 (0.01–4.03)	−12.0 (−30.0 to +8.9)	0.268

‡ The primary composite endpoint included major cannulation-related complications as predefined in the Methods. Patients experiencing more than one component were counted only once in the composite endpoint. In the DFC group, the three patients with arterial cannulation-site bleeding were the same three patients who subsequently developed wound infection.

## Data Availability

The data presented in this study are available on request from the corresponding author due to privacy and ethical restrictions concerning patient-level clinical information.

## Abbreviations

The following abbreviations are used in this manuscript:

ADG	Axillary Dacron Graft cannulation
BSA	Body Surface Area
CI	Confidence Interval
CPB	Cardiopulmonary Bypass
DFC	Direct Femoral Cannulation
ECMO	Extracorporeal Membrane Oxygenation
ICU	Intensive Care Unit
IABP	Intra-Aortic Balloon Pump
ISHLT	International Society for Heart and Lung Transplantation
KDIGO	Kidney Disease: Improving Global Outcomes
LVAD	Left Ventricular Assist Device
LVEF	Left Ventricular Ejection Fraction
OR	Odds Ratio
PGD	Primary Graft Dysfunction
SD	Standard Deviation
VA-ECMO	Veno-Arterial Extracorporeal Membrane Oxygenation
